# Flow cytometry and targeted immune transcriptomics identify distinct profiles in patients with chronic myeloid leukemia receiving tyrosine kinase inhibitors with or without interferon-α

**DOI:** 10.1186/s12967-019-02194-x

**Published:** 2020-01-03

**Authors:** Raquel Alves, Stephanie E. B. McArdle, Jayakumar Vadakekolathu, Ana Cristina Gonçalves, Paulo Freitas-Tavares, Amélia Pereira, Antonio M. Almeida, Ana Bela Sarmento-Ribeiro, Sergio Rutella

**Affiliations:** 1 Laboratory of Oncobiology and Hematology and University Clinic of Hematology/Faculty of Medicine, University of Coimbra (FMUC), Coimbra, Portugal; 2 Coimbra Institute for Clinical and Biomedical Research (iCBR) - Group of Environment Genetics and Oncobiology (CIMAGO), FMUC, Coimbra, Portugal; 3 Center for Innovative Biomedicine and Biotechnology (CIBB), University of Coimbra, Coimbra, Portugal; 4 John van Geest Cancer Research Centre, School of Science and Technology, Nottingham Trent University, Clifton Campus, Nottingham, NG11 8NS UK; 5 Clinical Hematology Department, Centro Hospitalar Universitário de Coimbra (CHUC), Coimbra, Portugal; 6 Internal Medicine Service, Hospital Distrital da Figueira da Foz (HDFF), Figueira da Foz, Portugal; 7 Hospital da Luz, Lisbon, Portugal; 8 CIIS (Centro de Investigação Interdisciplinar em Saúde, Universidade Católica Portuguesa de Lisboa), Lisbon, Portugal; 9 Centre for Health, Ageing and Understanding Disease (CHAUD), School of Science and Technology, Nottingham Trent University, Nottingham, UK

**Keywords:** Chronic myeloid leukemia, Interferon, Immunotherapy, Gene expression profiling, Immune monitoring

## Abstract

**Background:**

Tumor cells have evolved complex strategies to escape immune surveillance, a process which involves NK cells and T lymphocytes, and various immunological factors. Indeed, tumor cells recruit immunosuppressive cells [including regulatory T-cells (Treg), myeloid-derived suppressor cells (MDSC)] and express factors such as PD-L1. Molecularly targeted therapies, such as imatinib, have off-target effects that may influence immune function. Imatinib has been shown to modulate multiple cell types involved in anti-cancer immune surveillance, with potentially detrimental or favorable outcomes. Imatinib and other tyrosine kinase inhibitors (TKIs) in chronic myeloid leukemia (CML) have dramatically changed disease course. Our study aimed to characterize the different populations of the immune system in patients with CML affected by their treatment.

**Methods:**

Forty-one patients with CML [33 treated with TKIs and 8 with TKIs plus interferon (IFN)-α] and 20 controls were enrolled in the present study. Peripheral blood populations of the immune system [referred to as the overview of immune system (OVIS) panel, Treg cells and MDSCs] and PD-1 expression were evaluated by flow cytometry. The immunological profile was assessed using the mRNA Pan-Cancer Immune Profiling Panel and a NanoString nCounter FLEX platform.

**Results:**

Patients receiving combination therapy (TKIs + IFN-α) had lower numbers of lymphocytes, particularly T cells [838/µL (95% CI 594–1182)] compared with healthy controls [1500/µL (95% CI 1207 – 1865), p = 0.017]. These patients also had a higher percentage of Treg (9.1%) and CD4^+^PD-1^+^ cells (1.65%) compared with controls [Treg (6.1%) and CD4^+^/PD-1^+^(0.8%); p ≤ 0.05]. Moreover, patients treated with TKIs had more Mo-MDSCs (12.7%) whereas those treated with TKIs + IFN-α had more Gr-MDSC (21.3%) compared to controls [Mo-MDSC (11.4%) and Gr-MDSC (8.48%); p ≤ 0.05]. CD56^bright^ NK cells, a cell subset endowed with immune-regulatory properties, were increased in patients receiving TKIs plus IFN-α compared with those treated with TKIs alone. Interestingly, serum IL-21 was significantly lower in the TKIs plus IFN-α cohort. Within the group of patients treated with TKI monotherapy, we observed that individuals receiving 2nd generation TKIs had lower percentages of CD4^+^ Treg (3.63%) and Gr-MDSC (4.2%) compared to patients under imatinib treatment (CD4^+^ Treg 6.18% and Gr-MDSC 8.2%), but higher levels of PD-1-co-expressing CD4^+^ cells (1.92%).

**Conclusions:**

Our results suggest that TKIs in combination with IFN-α may promote an enhanced immune suppressive state.

## Background

Chronic myeloid leukemia (CML) is a clonal myeloproliferative disorder characterized by the presence of the oncogenic *BCR*-*ABL1* fusion gene derived from the reciprocal translocation of the long arms of chromosome 9 and chromosome 22 [1]. Disease course is typically triphasic, with the majority of patients presenting in the relatively stable chronic phase. However, if left untreated, patients with chronic-phase CML progress to accelerated phase and ultimately to blast crisis, which is invariably fatal [2].

The discovery of the unique molecular aberration of CML allowed the development of targeted therapies with tyrosine kinase inhibitors (TKIs), which revolutionized the management of CML in the late 1990s, offering the prospect of long-term disease control and near-normal life expectancy [3, 4]. Outside of clinical trials, three TKIs have been approved as front-line treatment for chronic-phase CML, i.e., imatinib, nilotinib, and dasatinib [1]. Although response rates are excellent, between 10 and 15% of CML patients fail to achieve adequate responses to multiple TKIs, due to the development of either resistance or intolerance. Patients with the deepest responses might be eligible for treatment interruption, given the observation that up to 40% of them remain in remission following TKI cessation [5]. Until the advent of TKIs, interferon (IFN)-α was used as standard therapy for chronic-phase CML. Interestingly, the upfront administration of TKIs and IFN-α, followed by low-dose IFN-α maintenance, enabled a high rate of imatinib discontinuation in CML patients in major molecular response (MMR) [6].

During tumor development, cancer cells evolve complex strategies to elude immune surveillance, a process aimed at restraining cancer cell proliferation and involving multiple cell types, such as natural killer (NK) cells and T lymphocytes, and numerous immune factors, such as IL-2, tumor necrosis factor (TNF)-α and IFN-γ [7]. Furthermore, cancer cells can recruit immunosuppressive cells, such as tumor-associated macrophages (TAM), regulatory T cells (Treg) and myeloid-derived suppressor cells (MDSCs) [8], and express or secrete immunosuppressive factors such as indoleamine 2,3-dioxygenase-1 (IDO1), and programmed death-ligand 1 (PD-L1) [9], all of which promote dysfunctional immune responses and shape a highly suppressive tumor microenvironment, ultimatey leading to exhaustion and/or apoptosis of PD-1-expressing cells via the activation of the PD-L1 signalling pathway [8, 10, 11]. CML promotes a highly immune-suppressive tumor microenvironment, by favoring lymphocyte anergy or exhaustion, and inducing the expansion of Treg cells and MDSCs [12, 13]. It has been shown that targeted anti-cancer therapies with TKIs may also have off-target or immune-mediated effects. For instance, imatinib modulates the function of multiple cell types involved in anti-cancer immune responses, with potentially detrimental as well as favorable outcomes [14]. The immunological effects of TKIs thus far described are diverse and include M2 reprogramming of TAMs [15]; inhibition of dendritic cell (DC) recovery [16] and effector cytokine production by CD4^+^ T cells [17]; reduction of IgM-producing memory B cells [18]; T helper 1 (Th1) polarization [19]; triggering of NK function [20, 21]; down-regulation of IDO1 [22]; normalization of MDSC numbers [23] and impairment of Treg function [24].

The immune changes induced by TKIs and IFN-α in patients with CML have not been investigated previously and have important translational implications to optimize clinical trials of TKI discontinuation. Herein, we profiled the peripheral immunome of CML patients treated with TKIs alone or in combination with IFN-α. We used the Overall Immune System (OVIS) staining panel for the flow cytometric assessment of key immune modulatory cell subsets, including Treg cells and MDSCs, and to quantify PD1 expression on T cells [25]. Additionally, we evaluated the blood immune transcriptome and we identified changes in immune gene expression profiles in patients treated with TKIs either alone or in combination with IFN-α. Taken together, our results suggest that TKIs in combination with IFN-α may promote an enhanced immune suppressive state in patients with CML.

## Methods

### Study population

Sixty-one subjects were enrolled in the present study (41 patients with CML and 20 healthy controls). The participants were recruited at Centro Hospitalar Universitário de Coimbra (CHUC) and Hospital Distrital da Figueira da Foz (HDFF, EPE), Portugal. Patients were grouped according to the specific treatment allocated (TKIs alone or TKIs plus IFN-α). Clinical and biological characteristics are summarized in Table 1. Treatment response criteria were defined according to the European Leukemia-Net (ELN) guidelines [1]. In the TKI group, 26 patients were classified as optimal responders and seven as a warning or failure. In the TKI plus IFN-α group, seven patients were classified as optimal responders and one patient as a warning. The study was conducted in accordance with the Helsinki Declaration, and all participants provided informed consent for participation prior to enrolment. The Ethics Committee of the Faculty of Medicine (University of Coimbra, Portugal) approved all research procedures.

**Table 1:** Biodemographic and clinical characteristics of patients and controls

Characteristics	CML patients		Controls (*n *= 20)
	TKI (*n *= 33)	TKI + IFN-α (*n *= 8)	
Demographic features			
Gender (%)			
Male	18 (54.5)	4 (50.0)	7 (35.0)
Female	15 (45.5)	4 (50.0)	13 (65.0)
Age (years)			
Median	63	50	58
Range	37–84	34–62	30–89
Clinical features			
Age at diagnosis (years)			
Median	50	42	
Range	24–78	25–60	
Time of disease (years)			
Median	11.2	3.4	
Range	1.3–22.7	2.1–24.1	
Scoring systems			
Sokal score	(*n *= 32)	(*n *= 7)	
Low risk (%)	13 (40.6)	4 (57.1)	
Intermediate risk (%)	13 (40.6)	1 (14.3)	
High risk (%)	6 (18.8)	2 (28.6)	
Euro score	(*n *= 32)	(*n *= 7)	
Low risk (%)	14 (43.8)	5 (71.4)	
Intermediate risk (%)	17 (53.2)	2 (28.6)	
High risk (%)	1 (3.0)	–	
Eutos score	(*n *= 33)	(*n *= 7)	
Low risk (%)	27 (81.8)	1 (14.3)	
High risk (%)	6 (18.2)	6 (85.7)	
Type of TKI			
Imatinib (%)	26 (78.8)	6 (75.0)	
Nilotinib (%)	1 (3.0)	2 (25.0)	
Dasatinib (%)	2 (6.1)	–	
Bosutinib (%)	3 (9.1)	–	
Ponatinib (%)	1 (3.0)	–	

### Overview of immune system (OVIS) flow cytometry panel

Peripheral blood was collected into EDTA Vacutainers. We transferred 100 µL of whole blood into Trucount™ tubes (BD Biosciences) using reverse pipetting. Cells were stained using a 10-color panel, containing fluorescently labeled monoclonal antibodies (mAbs) specific for the major immune cell populations. The OVIS panel included the following: anti-CD8 (FITC), anti-CD19 (PE), anti-CD28 (ECD), anti-CD56 (PE-Cy5), anti-CD3 (PE-Cy7), anti-CD45RA (APC), anti-CD14 (Alexa Fluor-700), anti-CD27 (APC eFluor-780), anti-CD45 (Pacific Blue), and anti-CD4 (Krome Orange) mAbs. After a 15-min incubation at room temperature, erythrocytes were lysed by BD Pharm Lyse™ reagent. Cells were run through a Gallios™ flow cytometer (Beckman Coulter), and data were analysed with the Kaluza Software (Beckman Coulter). The number of cells per microliter of whole blood was calculated as described by the manufacturer. For the Trucount method, 50 µL of mouse WB were added into Trucount tubes and processed *as per* the manufacturer’s protocol, except for the lysis buffer used.

### Isolation of peripheral blood mononuclear cells (PBMCs)

Peripheral blood mononuclear cells (PBMCs) were used for Treg and MDSC evaluation. PBMCs were separated from whole blood using density gradient centrifugation on Ficoll-Hypaque (GE Healthcare) according to the manufacturer’s protocol. After isolation, one aliquot of cells was used immediately, and the remaining aliquot was frozen (10 × 10^6^ cells/vial) for Treg studies.

### Regulatory T cell (Treg) assessment

Frozen PBMCs were thawed following the Cellular Technology Limited protocol (available online at http://www.immunospot.com). PBMCs were rested in RPMI-1640 supplemented with CTL-Wash™ for 2 h at 37 °C before staining with the following mAbs in the Treg panel: anti-PD-1 (FITC), anti-ICOS (PE), anti-CD3 (ECD), anti-CD25 (PE-Cy5), anti-CD39 (PE-Cy7), anti-CD8 (Alexa Fluor 700), anti-CD127 (APC eFluor 780), anti-CD4 mAbs (Krome Orange) and anti-FoxP3 (eFluor 660). A LIVE/DEAD™ Fixable Violet solution was used to exclude dead cells from the analysis. Briefly, 1 × 10^6^ cells were incubated for 10 min at 4 °C with FcR blocking reagent. After washing with PBS, PBMCs were stained for cell surface markers at room temperature for 10 min. The LIVE/DEAD™ Fixable Violet solution dye was then added, and cells were incubated for 30 min at room temperature. The FoxP3 Fix/Perm Kit was used for intracellular staining of FoxP3 according to the manufacturer’s protocol.

### Myeloid-derived suppressor cell (MDSC) evaluation

Immediately after isolation, 1 × 10^6^ PBMCs were stained with the MDSC antibody panel, which included anti-CD11b (PE), anti-CD33 (PE-Cy5), anti-CD15 (PE-Cy7), anti-arginase-1 (Alexa Fluor 700), and anti-CD45 (Pacific Blue) mAbs. Briefly, cells were incubated for 10 min at 4 °C with FcR blocking reagent. After washing with PBS, PBMCs were stained for cell surface markers at room temperature for 15 min in the dark. Cells were then fixed and permeabilized with the Fix/Perm solution for 30 min at room temperature in the dark. After a further washing step, cells were stained with anti-arginase-1 mAbs for 15 min at room temperature in the dark.

### Targeted immune gene expression profiling

We used the nCounter™ FLEX platform (NanoString Technologies Inc., Seattle, WA) to assess immune transcriptomic profiles in patient PBMCs [26]. The nCounter™ analysis system is a robust and highly reproducible method for detecting the expression of up to 800 genes in a single reaction with high sensitivity and linearity across a broad range of expression levels [27]. It is based on digital detection and direct molecular barcoding of individual target molecules through the use of a unique probe pair carrying 35- to 50-base target-specific sequences. This technology allows for direct multiplexed measurements of gene expression from a low amount of mRNA (25 to 300 ng) without the need for amplification by PCR. The RNA Pan-Cancer Immune Profiling Panel™, which includes 770 genes (109 cell surface markers for 24 immune cell types, 30 cancer-testis antigens, > 500 immune response genes, and 40 reference genes), was used in our experiments. Digital images were processed within the nCounter Digital Analyzer™ instrument, and the reporter probe counts, i.e., the number of times the color-coded barcode for that gene is detected, were tabulated in a comma-separated value (CSV) format for data analysis with the nSolver™ software package. The analysis software automatically performs quality controls, normalization, data analysis and creates reports with the options of performing advanced analyses, including pathway applications [28]. The nCounter Advanced Analysis module (version 2.0.115) was used to calculate the relative abundance of immune cell types. The total lymphocyte score was defined as the average of the B cell, T cell, CD45, macrophage and cytotoxic T-cell scores. The other relative abundance scores were calculated by subtracting the total lymphocyte score from each cell type score. For instance, a NK-cell score will measure the relative abundance of NK cells within the total immune population. Each score will increase by 1 when NK cells double their frequency relative to the 5 immune populations defining the total lymphocyte score.

### Measurement of serum IL-21

Serum was harvested after the commencement of treatment with either TKIs alone (n = 20 patients) or with TKIs and IFN-α (n = 8 patients) and from 12 healthy controls. IL-21 was quantitated using commercially available reagents (IL-21 LEGEND MAX™ Human ELISA kit; BioLegend, San Diego, CA; sensitivity: 4.2 pg/mL).

### Statistical analyses

Dependent variables were logarithmically transformed to achieve an approximation to a normal distribution and to reduce heterogeneity. We tested the effect of the independent variables on the measured parameters using linear models (LM). For each dependent variable, multiple pairwise comparisons were performed using sequential Bonferroni correction. Model validation was performed, for each LM, on the residuals by checking heteroscedasticity, normality, and influential observations. The results are expressed as estimated mean and 95% confidence intervals (CI) unless otherwise stated. For correlation analysis, the nonparametric Spearman rank test was used. All statistical comparisons were considered significant when *p* values were < 0.05. Statistical analyses were performed using the IBM-SPSS^®^ software package, version 22.

## Results

### The overview of immune system (OVIS) analysis highlights differences between treatment groups

In order to evaluate whether treatment with TKIs, either alone or in combination with IFN-α, had an impact on immune cell populations in patients with CML, we initially assessed the frequency and absolute numbers of leukocyte subsets and immune cell populations using the OVIS antibody panel (Additional file 1 and Table 2) [25]. The degree of peripheral lymphopenia was higher in CML patients receiving TKIs plus IFN-α [1140/µL of blood (95% CI 811–1603)] compared with individuals receiving TKIs only [1853/µL (95% CI 1567–2191); *p *= 0.039]. Not unexpectedly, patients treated with TKIs plus IFN-α had lower lymphocyte counts when compared with healthy controls [2059/µL (95% CI 1660–2554; *p *= 0.014)]. By contrast, no statistically significant differences were observed in granulocyte and monocyte counts (Table 2).

**Table 2:** Overview of immune system (OVIS)

Cell populations	Controls (*n *= 20)		CML patients						*p* value*#*
	Mean	95% CI	TKIs (*n *= 33)			TKIs plus IFN-α (*n *= 8)			
			Mean	95% CI	*p* value	Mean	95% CI	*p* value	
Granulocytes	3971.9	(3195.1–4937.6)	3752.5	(3167.6–4445.3)	1.000	4051.7	(2872.1–5715.9)	1.000	1.000
Monocytes	328.7	(261.2–413.6)	381.5	(319.0–456.2)	0.930	466.4	(324.4–670.7)	0.326	0.973
CD45^low^	140.9	(104.5–189.9)	*64.8*	*(51.4–81.8)*	*0.001*	*56.1*	*(35.0–90.0)*	*0.005*	1.000
Lymphocytes	2059.4	(1660.5–2554.1)	1852.7	(1566.9–2190.8)	1.000	*1140.3*	*(811.3–1602.6)*	*0.014*	*0.039*
B cells	241.9	(176.2–332.1)	147.9	(115.6–189.3)	0.052	171.6	(104.0–283.1)	0.751	1.000
T cells	1500.3	(1207.0–1864.9)	1324.3	(1118.0–1568.7)	1.000	*837.9*	*(594.1–1181.9)*	*0.017*	0.060
CD4^+^	1016.4	(813.2–1270.4)	758.0	(637.1–901.7)	0.104	*504.7*	*(354.7–718.1)*	*0.004*	0.105
Naive	358.5	(264.0–486.9)	230.7	(181.8–292.7)	0.067	*173.1*	*(103.2–290.3)*	*0.048*	0.573
Central memory (_CM_)	344.3	(271.2–437.1)	*238.3*	*(197.9–286.9)*	*0.047*	*183.8*	*(122.8–275.1)*	*0.026*	0.475
Effector memory (_EM_)	13.7	(6.9–27.1)	18.2	(10.7–31.0)	0.791	7.1	(2.2–22.6)	0.596	0.311
EM CD45 RA^+^ (_EMRA_)	4.2	(2.1–8.1)	*11.9*	*(7.0–20.1)*	*0.044*	4.6	(1.5–14.3)	0.988	0.289
CD8^+^	207.1	(152.5–281.4)	327.7	(258.2–416.0)	0.055	193.2	(119.0–313.7)	0.968	0.132
Naive	42.9	(29.1–63.2)	51.7	(38.2–69.8)	0.730	57.2	(31.0–105.5)	0.708	0.952
Central memory (_CM_)	39.5	(28.7–54.3)	48.1	(37.5–61.6)	0.595	35.6	(21.5–58.9)	0.936	0.536
Effector memory (_EM_)	5.2	(2.9–9.4)	*19.7*	*(12.4–31.2)*	*0.002*	10.5	(4.1–26.8)	0.411	0.454
EM CD45 RA^+^ (_EMRA_)	13.3	(7.5–23.6)	*74.7*	*(47.7–117.0)*	*0.001*	23.0	(9.3–57.2)	0.566	0.060
CD4^+^/CD8^+^	228.3	(168.0–310.2)	*126.3*	*(99.5–160.3)*	*0.009*	*82.0*	*(50.5–133.1)*	*0.002*	0.253
NK cells	243.4	(175.1–338.3)	233.4	(180.7–301.6)	1.000	159.1	(94.6–267.7)	0.517	0.573
CD56^Dim^	243.3	(174.0–340.2)	266.2	(205.1–345.6)	0.906	135.8	(80.0–230.7)	0.159	0.067
CD56^Bright^	11.2	(7.3–17.3)	6.3	(4.5–8.8)	0.092	28.6	(14.5 - 56.5)	0.060	*0.001*
CD56^+^ T cells	60.6	(36.9–99.6)	41.4	(28.1–61.0)	0.697	*8.8*	*(4.0*–*19.4)*	*0.001*	*0.002*
CD4^+^	14.0	(8.7–22.5)	7.9	(5.4–11.5)	0.154	*2.4*	*(1.0*–*5.7)*	*0.002*	*0.039*
CD8^+^	15.8	(9.3–26.8)	19.0	(12.4–29.1)	0.848	6.6	(2.5–17.3)	0.259	0.119
CD4^+^/CD8^+^	22.0	(13.4–36.3)	*9.8*	*(6.6*–*14.7)*	*0.038*	*3.6*	*(1.4*–*8.9)*	*0.003*	0.111

*p* value: statistical comparison *vs* control*. p* value *#* statistical comparison between TKIs and TKIs plus IFN-α. Cell populations with p < 0.050 are highlighted in italic

**Fig. 1:**
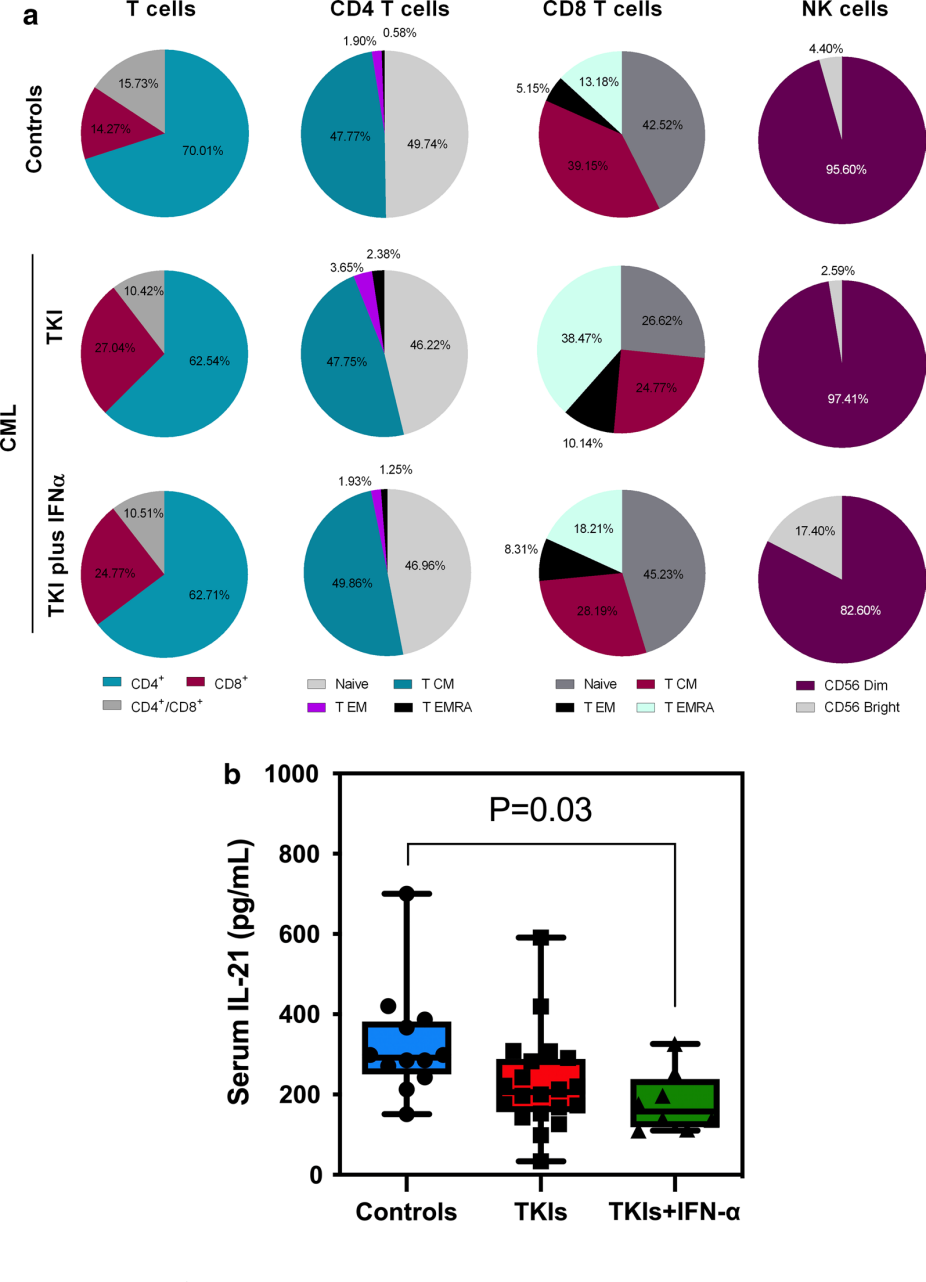
Frequency of immune cell types in patients with CML receiving TKIs, either alone or in combination with IFN-α, and in healthy controls. **a** Pie charts summarizing the distribution of T cells, CD4^+^ and CD8^+^ major subsets, and NK cells in the blood of CML patients and healthy controls. T_CM_ = central memory T cell; T_EM_ = effector memory T cell; T_EMRA_ = terminally differentiated, effector memory T cell. **b** Serum IL-21 levels in a subgroup of patients receiving either TKIs alone of TKIs in combination with IFN-α, and in healthy controls. Results were compared with one-way ANOVA with Tukey’s multiple comparisons test

The numbers of circulating T cells and B cells, as defined by their expression of CD3 and CD19, respectively, were not significantly different in the TKIs-only group compared with controls. Interestingly, the count of CD3^+^ T cells was significantly lower in the TKIs plus IFN-α group [838/µL (95% CI 594–1182)] compared with controls [1500/µL (95% CI 1207–1865), p = 0.017]. We next categorized T-cell populations based on CD4 and CD8 expression and we also quantified functionally distinct naïve and memory CD4^+^ and CD8^+^ subsets (i.e., naïve T cells [T_N_], central memory T cells [T_CM_], effector memory T cells [T_EM_] and terminally-differentiated effector memory T cells [T_EMRA_]) using well established combinations of mAbs (Additional file 1) [29]. Although the CD4^+^ T-cell compartment was marginally affected by treatment with TKIs alone, we observed a reduction of overall CD4^+^ T-cell counts (p = 0.004), naïve CD4^+^ T cells (p = 0.048) and T_CM_ cells (p = 0.026; Fig. 1a) in patients treated with TKIs and IFN-α compared with controls. Treatment with TKIs also translated into an increase of CD8^+^ T_EM_ and CD8^+^ T_EMRA_ compared with controls (p = 0.002 and p = 0.001, respectively). Finally, the absolute number of double-positive CD4^+^CD8^+^ T cells was significantly lower in both treatment groups compared with controls, an effect which was more pronounced in the TKIs plus IFN-α group (p = 0.002; Table 2).

The number of NK cells was similar in blood samples from CML patients and controls. However, CD56^bright^ NK cells were significantly increased in patients receiving TKIs plus IFN-α compared with those treated with TKIs alone (p = 0.001; Fig. 1). Interestingly, serum IL-21 levels were significantly lower in patients treated with TKIs and IFN-α compared with controls (Fig. 1b). We also observed a trend towards higher serum IL-21 levels in patients receiving TKIs only compared with the combination therapy group. NKT cells, defined as CD56-expressing CD3^+^ T cells, were significantly decreased in CML patients given TKIs plus IFN-α [8.8/µL (95% CI 4.0–19.4)] relative to controls [60.6/µL (95% CI 36.9–99.6), p = 0.001]. When analyzing CD4- and CD8-coexpressing CD56^+^ T cells, we observed that the CD4^+^ subset was predominantly reduced in patients receiving combination treatment with TKIs and IFN-α.

Taken together, these experiments suggest that the immune profile of patients treated with TKIs alone shows a greater similarity to that of age-matched healthy controls compared to the peripheral immunome of patients receiving TKIs and IFN-α. Furthermore, patients given combination therapy showed a higher degree of lymphopenia, affecting both naïve and memory CD4^+^ T cells.

### Treatment with TKI plus IFN-α increases Treg cells in CML patients

**Fig. 2:**
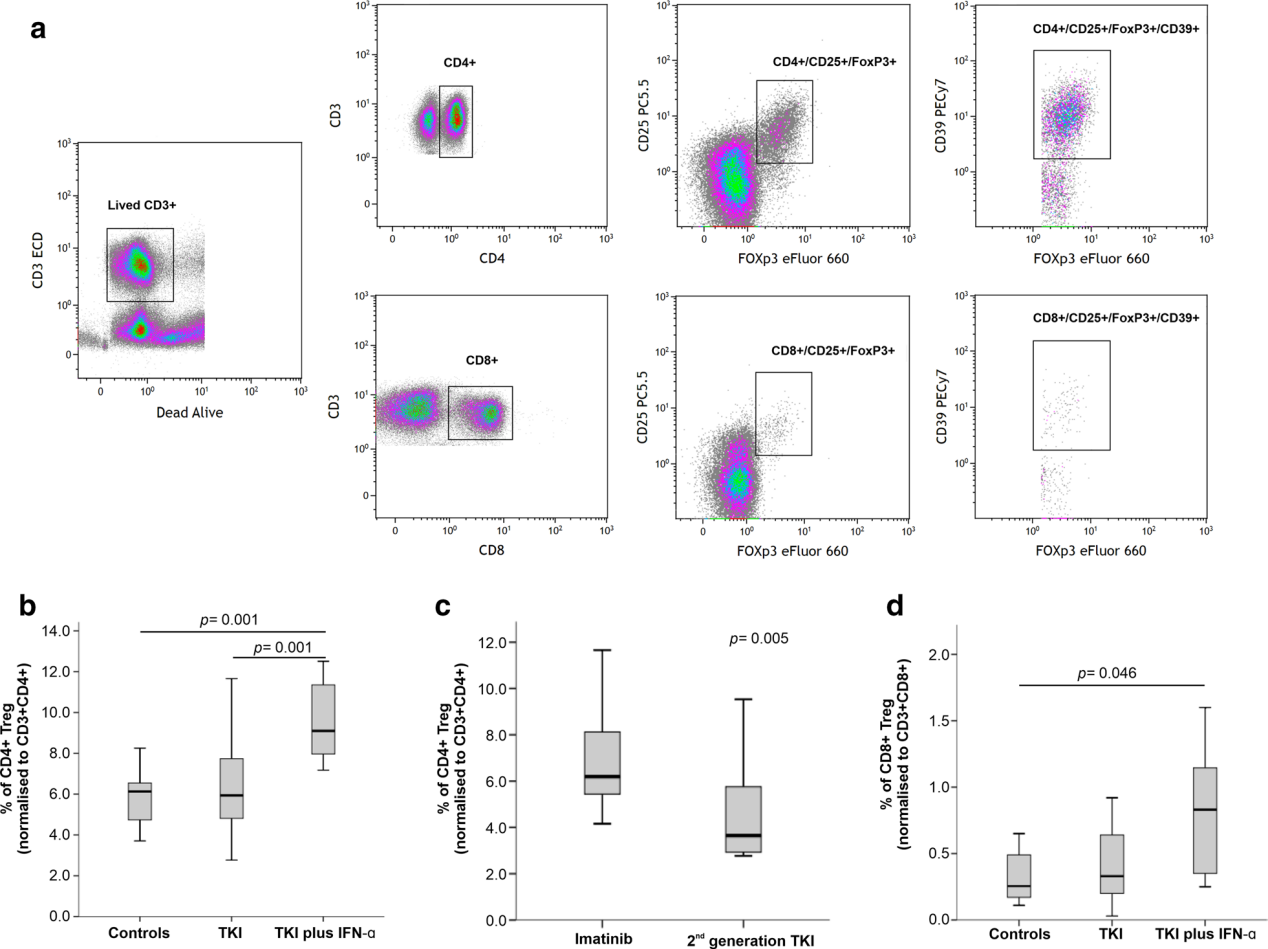
Regulatory T cells (Treg) in patients with CML receiving TKIs, either alone or in combination with IFN-α, and in healthy controls. **a** Gating strategy used to identify blood Treg cells within the CD4^+^ and CD8^+^ T-cell compartment. **b** Percentage of CD4^+^ Treg cells in different patient groups (TKIs group, n = 33; TKIs plus IFN-α group, n = 8) and in healthy controls (n = 20). **c** Frequency of CD4^+^ Treg cells in patients with CML receiving imatinib (n = 26) or 2nd generation TKIs (n = 7). **d** Frequency of CD8^+^ Treg cells in patients with CML receiving TKIs, either alone or in combination with IFN-α, and in healthy controls. The *p* values in the figure reflect statistically significant differences among study groups

We next measured Treg cells, defined by either a CD3^+^CD4^+^CD25^++^FoxP3^+^ or a CD3^+^CD8^+^CD25^++^FoxP3^+^ phenotype, in CML patients receiving TKIs alone or TKIs plus IFN-α, and in healthy controls. The percentage of *bona fide* Treg cells was increased in approximately 50% of CML patients treated with TKIs plus IFN-α compared with patients given TKIs alone (p = 0.001) and with healthy controls (p = 0.001) (Fig. 2a). We then attempted to correlate Treg numbers with TKI generation in patients receiving this treatment modality alone (Additional file 2). Patients treated with imatinib (a 1st generation TKI) had a higher frequency of blood Treg cells compared with patients treated with 2nd generation TKIs (6.18% versus 3.63%; p = 0.005) (Fig. 2b). Interestingly, patients treated with TKIs plus IFN-α showed a 3.4-fold increase of CD8^+^ Treg cells compared with controls (p = 0.046; Fig. 2c). Using CD39 expression as a surrogate marker for Treg activation, we did not observe any differences in the activation status when comparing CML patients and controls (data not shown).

**Fig. 3:**
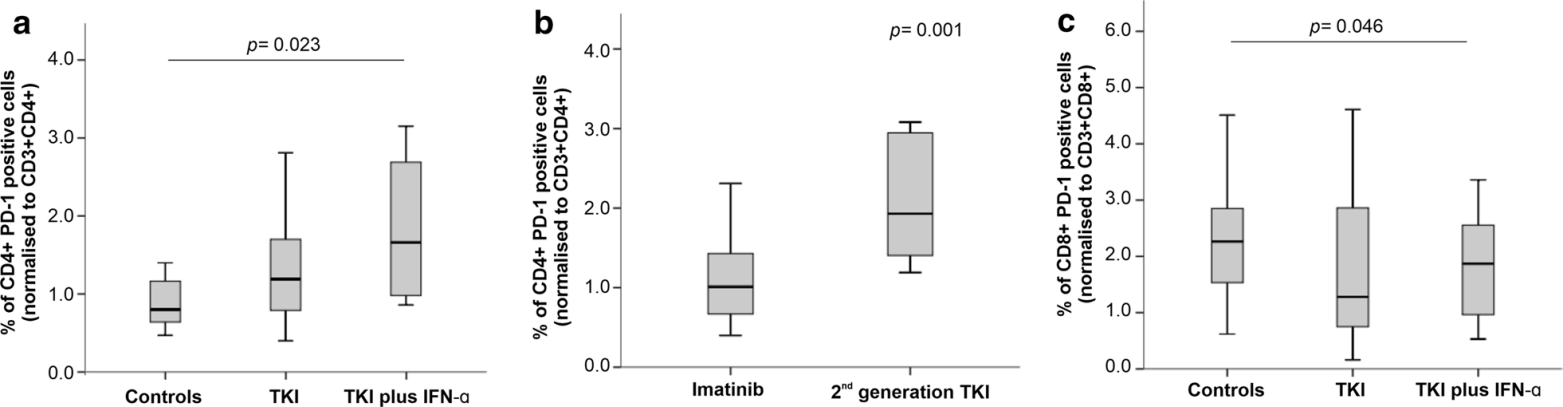
Programmed death receptor 1 (PD-1) expression on CD4^+^ and CD8^+^ T cells in patients with CML receiving TKIs, either alone or in combination with IFN-α, and in healthy controls. PD-1 expression on CD4^+^ (**a**, **b**) and CD8^+^ T cells (**c**) from CML patients stratified by type of TKI used (imatinib or 2nd generation TKI). The *p* values in the figure reflect statistically significant differences among study groups

We also evaluated the expression of PD1 on both CD4^+^ and CD8^+^ T cells. As shown in Fig. 3, CD4^+^ T cells expressed higher levels of PD1 in both CML treatment groups, with a statistically significant difference being detected when comparing patients on TKIs plus IFN-α and controls (1.65% versus 0.8%; p = 0.023; Fig. 3a). When restricting our analysis to the TKIs group, we observed that patients treated with 2nd generation TKIs had higher percentages of PD1-expressing CD4^+^ T cells compared with patients receiving imatinib (1.92% versus 1.0%; p = 0.001) (Fig. 3b, Additional file 3). By contrast, we observed lower PD1 expression on the CD8^+^ T cells of patients treated with TKIs plus IFN-α (p = 0.046; Fig. 3c).

### MDSC levels are modulated by CML treatment

**Fig. 4:**
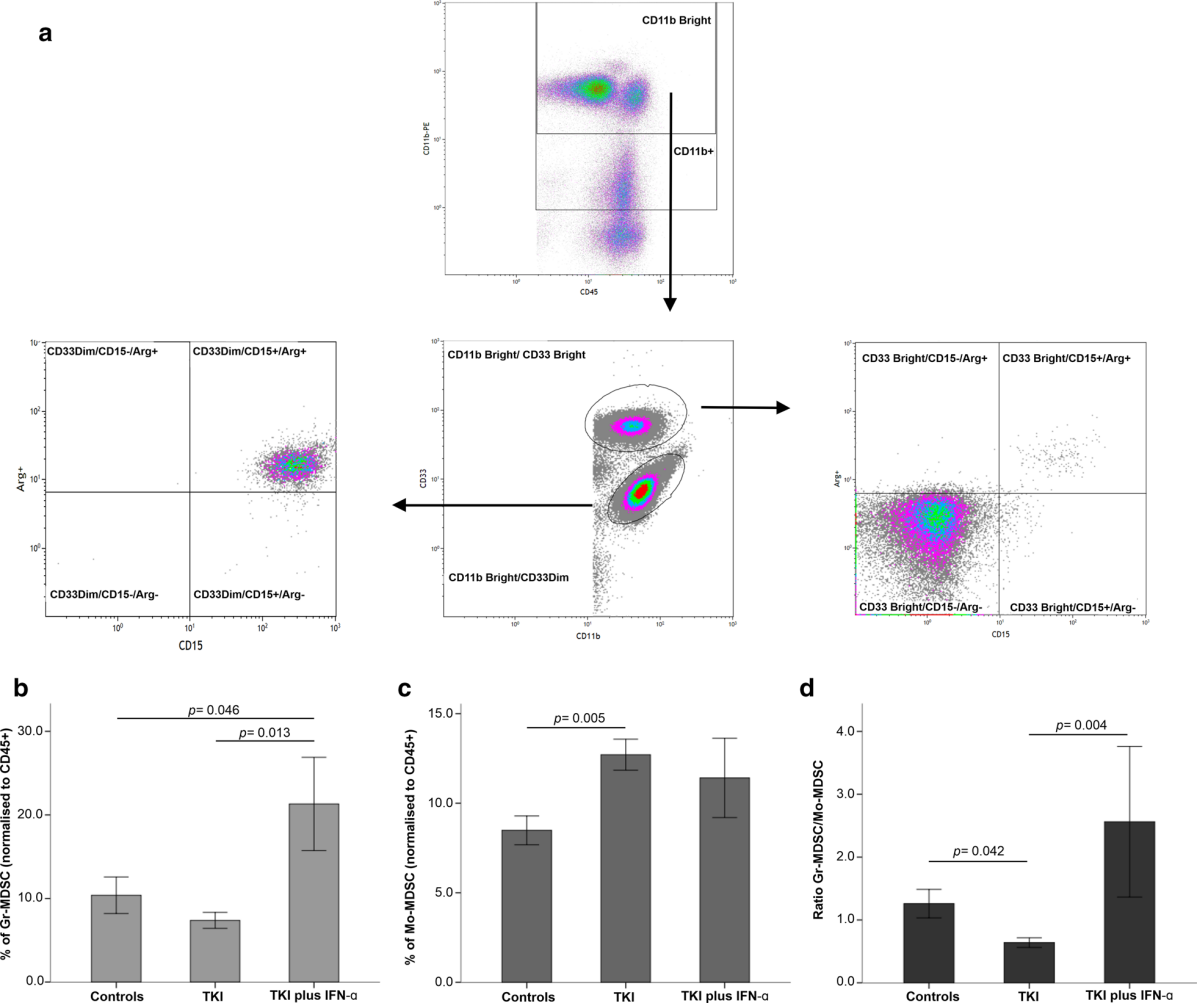
Frequency of myeloid-derived suppressor cells (MDSCs) in patients with CML receiving TKIs, either alone or in combination with IFN-α. **a** Gating strategy for the identification of granulocytic MDSCs (Gr-MDSCs: CD45^+^CD11b^bright^CD33^dim^CD15^+^Arg1^+^) and monocytic-like MDSCs (Mo-MDSCs: CD45^+^CD11b^bright^CD33^bright^CD15^neg^Arg1^neg^). The frequency of Gr-MDSCs (**b**), Mo-MDSCs (**c**) and the ratio of Gr-MDSCs to Mo-MDSCs (**d**) are shown in CML patients and in healthy controls. Results are summarized as the mean ± SEM. The *p* values in the figure reflect statistically significant differences among study groups

We next quantified granulocytic (Gr) MDSCs, defined as CD45^+^CD11b^bright^CD33^dim^CD15^+^Arg1^+^ cells, and monocytic-like (Mo) MDSCs, defined as CD45^+^CD11b^bright^CD33^bright^CD15^neg^Arg1^neg^ cells, in CML patients and controls. Gr-MDSC levels were reduced in patients treated with TKIs relative to controls, albeit differences failed to achieve statistical significance (Fig. 4a). Interestingly, patients receiving TKIs plus IFN-α had 21.3% blood Gr-MDSCs on average, a proportion that was significantly higher than that observed in the control group (p = 0.046) and in patients treated with TKIs only (p = 0.013; Fig. 4b). In contrast, the TKIs-only patient group had the highest average level of Mo-MDSCs (12.7%), followed by the TKIs plus IFN-α group (11.4%) and the control group (8.48%; p = 0.005; Fig. 4c). Finally, the ratio of Gr-MDSCs to Mo-MDSCs was 1.2 in healthy individuals, 0.63 in patients treated with TKIs only (p = 0.042), and 2.56 in patients treated with TKIs plus IFN-α (p = 0.004; Fig. 4d).

**Fig. 5:**
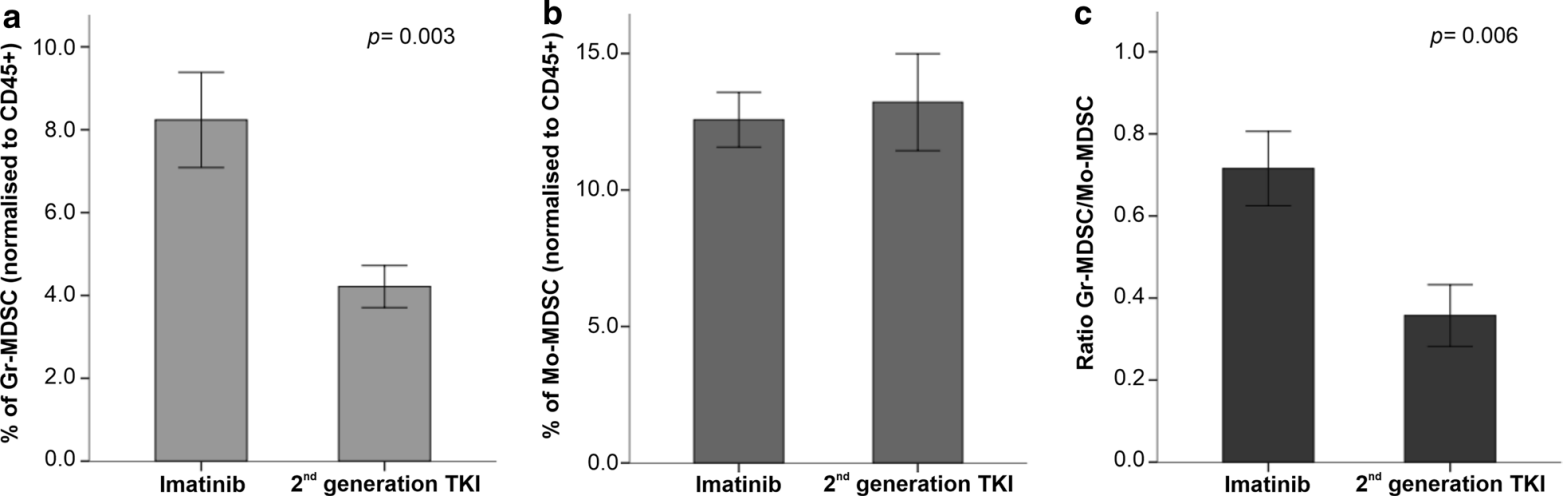
Frequency of myeloid-derived suppressor cells (MDSCs) in patients with CML receiving imatinib or 2nd generation TKIs. The frequency of Gr-MDSCs (**a**), Mo-MDSCs (**b**) and the ratio of Gr-MDSCs to Mo-MDSCs (**c**) are shown in CML patients treated with imatinib or with 2nd generation TKIs. Results are summarized as the mean ± SEM. The *p* values in the figure reflect statistically significant differences among study groups

When evaluating the impact of 1st generation and 2nd generation TKIs on MDSC levels, we observed that the proportion of Gr-MDSC was significantly lower in patients receiving 2nd generation TKIs (p = 0.003; Fig. 5 and Additional file 4). In contrast, Mo-MDSC levels were not affected. Although patient numbers are too low to allow definitive conclusions, it is interesting to note that two individuals treated with dasatinib showed the lowest levels of Gr-MDSCs and the highest levels of Mo-MDSCs (Additional file 4).

### Transcriptomic analyses identify distinct immune gene expression profiles in the blood of CML patients treated with TKI and IFN-α

**Fig. 6:**
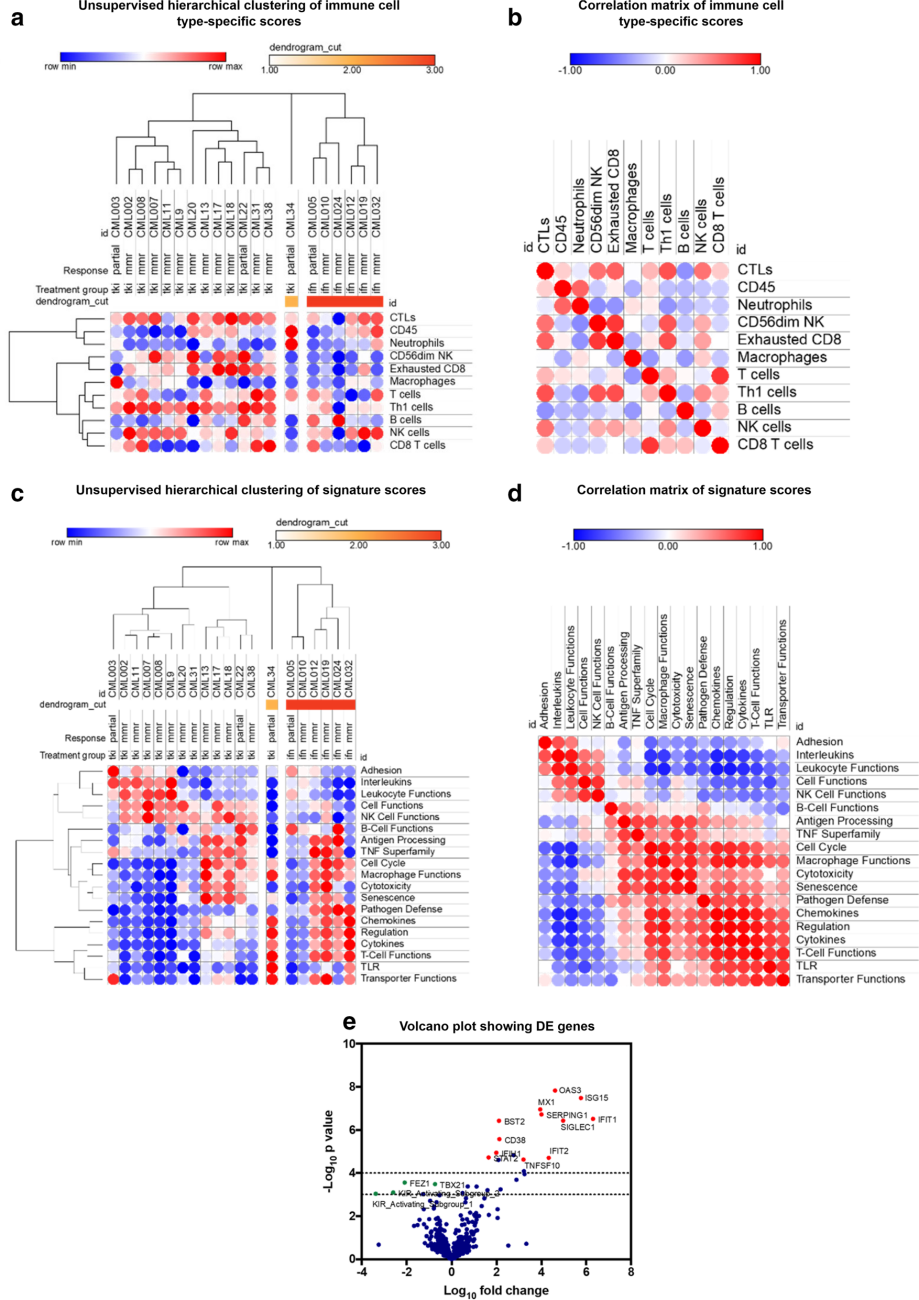
Gene expression profiling of blood samples from patients with CML receiving TKIs, either alone or in combination with IFN-α. **a** Unsupervised hierarchical clustering of immune cell type-specific scores in patients treated with IFN-α and TKIs or with TKIs alone. Data were analyzed and visualized using an on-line resource (Morpheus; Broad Institute, MA, USA). **b** Correlation matrix (Pearson correlation coefficients) of immune cell type-specific scores in patients treated with TKIs plus IFN-α or with TKIs alone. Dark red denotes high correlation, dark blue denotes anti-correlation and white denotes a lack of correlation. **c** Unsupervised hierarchical clustering of signature scores in patients treated with IFN-α and TKIs or with TKIs alone. **d** Correlation matrix (Pearson correlation coefficients) of signature scores in patients treated with TKIs plus IFN-α or with TKIs alone. Dark red denotes high correlation, dark blue denotes anti-correlation and white denotes a lack of correlation. **e** Volcano plot showing differentially expressed (DE) genes in patients receiving IFN-α and TKIs compared with patients treated with TKIs alone. The top DE genes (linear fold-change > 4 or < 2 with a *p* value < 0.01) are highlighted in red (up-regulated) and green (down-regulated). The dotted horizontal lines indicate *p* values < 0.05 and < 0.01

In a final set of experiments, we used the nCounter gene expression profiling platform to analyze the immune transcriptome of a subgroup of 20 CML patients from our initial cohort [30, 31]. Fourteen patients were assessed at various time points from the commencement of TKIs and six patients were assessed on IFN-α therapy. Figure 6a shows the results of unsupervised hierarchical clustering of the immune cell type-specific scores generated by the nSolver™ software. A detailed list of genes used to identify each immune cell subset is available from a previous publication [32]. Patients treated with a combination of TKIs and IFN-α expressed lower levels of transcripts encoding molecules known to be expressed on cytotoxic T lymphocytes, Th1 cells, B cells and KIR-expressing CD56^dim^ NK cells. In addition, exhausted CD8^+^ T cells were less represented in the blood of patients receiving TKIs plus IFN-α compared with patients treated with TKIs only. The transcriptomic profile of patient #34 was markedly different from that of the two major clusters of CML patients, insofar as neutrophil-specific mRNA species and CD45 mRNA were highly expressed. A correlation matrix of immune cell type-specific scores, which reflects the co-expression patterns of immune-related mRNAs detected in patient blood, is shown in Fig. 6b. Interestingly, the expression of markers for exhausted CD8^+^ T cells positively correlated with that of CD56^dim^ NK cells, predominantly representing KIR-expressing NK-cell populations [32]. We also analyzed signature scores which reflect the activation of relevant biological processes. As shown in Fig. 6c, our cohorts of CML patients could be clearly separated based on the expression profiles of specific gene modules. In particular, patients treated with a combination of TKIs plus IFN-α expressed lower levels of genes encoding NK function-associated molecules, interleukins and adhesion molecules compared with patients treated with TKIs alone. This finding is in agreement with the observed reduction of serum IL-21 levels in patients on combination therapy (Fig. 1b). In contrast, the expression levels of genes associated with macrophage function, pathogen defense, T-cell function and cytokine/chemokine production were higher after combination therapy. The correlation matrix of signatures scores allowed us to identify co-expression and mutual exclusivity patterns of the above gene modules (Fig. 6d). Not unexpectedly, differential expression (DE) analysis showed the induction of IFN pathway genes in patients receiving TKIs and IFN-α compared with patients treated with TKIs alone (Fig. 6e), including the over-expression of *MX1*, *ISG15*, *IFIT1* and *OAS3*. The full list of differentially expressed genes is provided in Additional file 5.

## Discussion

Several studies have demonstrated that the immunological landscape of the tumor may affect treatment response [33, 34]. Particularly in CML, the main treatment goal is to achieve and sustain deep molecular responses that could lead to TKI discontinuation and to a state of treatment-free remission [35]. Currently, approximately half of the patients with CML who discontinue TKI therapy relapse. In patients who achieve good clinical results, the success has generally been attributed to the re-activation of the immune system, which then effectively controls leukemia cell proliferation [36, 37].

The immunological profile of an individual patient is a dynamic process that is affected by several factors, including tumor cell characteristics, the tumor microenvironment and specific treatment modalities. Considering its susceptibility to immune system attack and the favorable results obtained with IFN-α in the pre-TKI era [38], CML qualifies as an ideal scenario for combination therapies with TKIs and IFN-α. This regimen has been shown to increase the rate of molecular responses in comparison with imatinib monotherapy [39]. Additionally, some authors have suggested the use of type I IFN at time of TKI discontinuation as a strategy to boost immune system responses [6, 40]. In our cohort of CML patients, treatment responses, as measured by *BCR*-*ABL* levels, were very satisfactory. However, the impact of long-term combination treatment on immune cell populations is presently unknown.

Herein, we present a comprehensive evaluation of the peripheral immunome of CML patients treated with TKI monotherapy or with TKIs plus IFN-α. Several immune subpopulations are reportedly increased at the time of diagnosis. However, the use of TKIs treatment has been shown to reduce these proportions to levels that are similar to those observed in healthy subjects [41]. Nonetheless, significant differences were found in immune cells associated with the disease as well as linked to the use of IFN in the therapeutic scheme. Antitumor effects of IFN-α are supported not only by the direct actions on tumor cells (inhibition of cell proliferation and induction of apoptosis) but also by immune stimulation (enhancing T-cell activation, promoting DC maturation and stimulating NK cell activity) [42, 43]. In accordance with this knowledge, we observed a higher count of CD56^bright^ NK cells in the combination group compared to TKIs in monotherapy. CD56^bright^ NK cells are considered to be regulatory NK cells that can exert beneficial or detrimental effects to the host, depending on the characteristics of the tissue microenvironment involved [44, 45]. Interestingly, an early increase of CD56^bright^ NK cells has been documented in patients with multiple sclerosis receiving immunotherapy with daclizumab, an anti-CD25 monoclonal antibody [46, 47]. We also detected significantly lower levels of serum IL-21 in CML patients receiving TKIs and IFN-α, compared with healthy controls and with patients on TKIs only. IL-21 priming has previously been reported to boost NK-cell maturation in vitro in synergy with IL-15 [48]. Our observation therefore reinforces the contention that combination therapy with TKIs and IFN-α may induce an enhanced immunosuppressive state by also promoting the expansion of immature CD56^bright^ NK cells.

The transcriptomic analysis of blood samples collected from CML patients allocated to different treatment modalities revealed high levels of genes encoding NK-function associated molecules (as KIR-expressing CD56^dim^ NK cells) and low levels of genes related to cytokine/chemokine production. By integrating immune cell quantification with high-dimensional flow cytometry and immune transcriptomic analyses, our study suggests that one possible mechanism of action for IFN-α may be related to the modulation of cytokine and chemokine production, therefore boosting adaptive immune responses. Some authors have reported that long-term exposure to imatinib and other TKIs promotes the expansion of circulating NK cells, a phenomenon which may favorably affect the outcome of TKI discontinuation [49, 50]. In our study, we did not detect any differences in NK-cell proportions in the TKI-only group, which were similar to those observed in the control population. Furthermore, we found that IFN-α treatment in combination with TKI therapy induces a significant reduction in CD56^+^ T cells.

CML as a chronic disease induces a state of immune dysfunction as well as T-cell exhaustion, mainly due to chronic stimulation of immune cells in an immunosuppressive microenvironment [41]. Several players may favor immune escape of cancer cells, including Treg cells and MDSCs, either directly or via the induction of inhibitory receptors on effector cells [51]. Treg cells play an essential role in sustaining immunological unresponsiveness against tumor-associated antigens [52]. In several neoplasms, high percentages of Treg cells at the time of diagnosis or during treatment have been associated with a poor prognosis, including in hematological malignancies [53]. Imatinib treatment may affect the function of Treg cells through the inhibition of IDO1 and by impairing the expression of FoxP3, thus leading to Treg cell apoptosis [14, 24]. During TKI therapy, a reduction of Treg proportions to values similar to those in healthy volunteers would be anticipated. Furthermore, IFN-α treatment reduced Treg numbers in patients with melanoma and renal cell carcinoma, tentatively attributable to the inhibition of IL-2 production which modulates Treg cell proliferation and activation [54]. Unexpectedly, the highest levels of Treg cells, both within the CD4 and the CD8 subset, were observed in the TKI plus IFN-α patient group in our study.

Current observations highlight the crosstalk between tumor cells, stroma and immune cells. An inflammatory microenvironment modulates normal myelopoiesis in favor of MDSCs, one of the most potent immunosuppressive cell subsets that may promote tumor progression. Modulation of Treg cells, up-regulation of PD-L1 and release of molecules able to affect immune effector cells are some of the most critical MDSC functions thus far reported [55]. In CML, an increase in MDSCs at diagnosis has been observed and these cells were shown to be derived from a tumoral clone, as confirmed by *BCR*-*ABL* expression [56]. According to current literature, both TKIs and IFN-α as monotherapy are able to reduce MDSC counts, probably as a result of maturation induction [33, 42]. Our data demonstrates that combinatorial therapy is associated with higher levels of Gr-MDSCs compared with TKI monotherapy. In contrast, the TKIs-only group showed the lowest levels of Gr-MDSCs but a significant increase of Mo-MDSCs. Chronic exposure to IFN-α in low doses may result in a suppressive environment through activation of MDSC cells [57]. The reduction on MDSC number by IFN-α, described by other authors, might be related to short-term treatment, and it is conceivable that long-term treatments would see the number of these cells increase again. Contrary to the low numbers expected, Stanojevic et al. [42] described that the long-term effects of IFN-α on MDSC levels may differ from the short-term effects, as they observed a recovery of MDSC numbers. Collectively, our results show that the association of IFN-α to TKI therapy may drive a more suppressive environment supported by higher levels of Treg cells and MDSCs as well as more CD4^+^PD1^+^ cells.

Another aspect explored in our study is whether different types of TKIs may induce peculiar immune profiles. Patients treated with imatinib showed higher levels of Gr-MDSCs and CD4^+^ Treg cells but lower proportions of PD1^+^ cells compared to patients given 2nd generation TKs. It has been shown that each TKI may differentially impact Treg, MDSC and PD1^+^ cells, a phenomenon that could be explained by the different kinases targeted by each of them rather than BCR-ABL [40, 41]. For instance, dasatinib also targets the RC kinase which plays an important role in T and B-cell activation and proliferation [9, 33]. In fact, our results support that changes of immune cell frequencies may be related to the specific TKI used for treatment. In this respect, patients receiving 2nd generation TKIs showed higher proportions of exhausted CD4^+^ T cells compared to patients receiving imatinib, an observation that could be accounted for by the inhibition of other signaling pathways.

## Conclusions

Within the constraints of important limitations, including the lack of functional data and the absence of experiments using primary bone marrow samples, our study highlights the occurrence of immune modulation in patients receiving combination therapy with TKIs plus IFN-α and it also documents an impact of specific TKIs on different immune cell populations. Although the results shown here need to be validated in a larger cohort of CML patients, the administration of IFN-α might be a valuable strategy to boost immune surveillance, to possibly eradicate leukemic stem cells and to support TKI discontinuation, if associated with careful monitoring of immunosuppressive cells.

## Supplementary information

**Additional file 1.** Gating strategy for the enumeration of peripheral blood populations of the immune system [referred to as the overview of immune system (OVIS) panel]. Leukocyte populations were initially identified based on CD45 expression and side scatter characteristics. CD14 was used as a monocytic marker (A), whereas B and T cells were defined based on the expression of CD19 and CD3, respectively (B). CD4 and CD8 subpopulations were further categorized using CD45RA, CD27 and CD28 staining. (C) NKT cells were identified based on CD56 and CD3 expression, and CD56^+^ T cells were further subdivided based on CD4 and CD8 expression.

**Additional file 2.** Frequency of Treg cells in patients with CML receiving imatinib or 2nd generation TKIs. Panels (A) and (B) summarize the frequency of CD4^+^ Treg cells in patients with CML receiving imatinib (n = 26) or 2nd generation TKIs (n = 1 nilotinib, n = 2 dasatinib, n = 3 bosutinib and n = 1 ponatinib). Panels (C) and (D) depict the frequency of CD8^+^ Treg cells in the same treatment categories. In the combination treatment group, 6 CML patients were treated with imatinib and 2 CML patients received nilotinib.

**Additional file 3.** Programmed death receptor 1 (PD-1) expression in patients with CML receiving imatinib or 2nd generation TKIs. Panels (A) and (B) summarize the frequency of PD-1-expressing CD4^+^ T cells in patients with CML receiving imatinib (n = 26) or 2nd generation TKIs (n = 1 nilotinib, n = 2 dasatinib, n = 3 bosutinib and n = 1 ponatinib). Panels (C) and (D) depict the frequency of PD-1-expressing CD8^+^ T cells in the same treatment categories. In the combination treatment group, 6 CML patients were treated with imatinib and 2 CML patients received nilotinib.

**Additional file 4.** Frequency of myeloid-derived suppressor cells (MDSCs) in patients with CML receiving imatinib or 2nd generation TKIs. Panels (A-C) and (B-D) summarize the frequency of Gr-MDSCs and Mo-MDSCs, respectively, in patients with CML receiving imatinib (n = 26) or 2nd generation TKIs (n = 1 nilotinib, n = 2 dasatinib, n = 3 bosutinib and n = 1 ponatinib). In the combination treatment group, 6 CML patients were treated with imatinib and 2 CML patients received nilotinib.

**Additional file 5.** List of differentially expressed immune genes when comparing CML patients treated with TKIs plus IFN-α and patients receiving TKIs alone. The differentially expressed genes (fold change > 4 or < 2) are ranked by corrected *p* value. Data were analyzed using the nSolver™ software package, version 4.0 (NanoString Technologies Inc., Seattle, WA).

## Data Availability

The datasets used and/or analyzed during the current study are available from the corresponding author on reasonable request and for legitimate scientific use.

## Abbreviations

CML: chronic myeloid leukemia
CTL: cytotoxic T lymphocyte
DC: dendritic cell
IDO: indoleamine 2,3-dioxygenase
IFN: interferon
IFT1: interferon-induced protein with tetratricopeptide repeats 1
IL: interleukin
IS: immune system
ISG15: interferon-stimulated gene 15
KIR: killer cell immunoglobulin like receptor
MDSCs: myeloid-derived suppressor cells
MMR: major molecular response
MX1: MX dynamin like GTPase 1 gene
NK: natural killer
OAS3: 2′-5′-oligoadenylate synthetase 3
OVIS: overview of immune system
PBMCs: peripheral blood mononuclear cells
PD-1: programmed death receptor 1
PD-L1: programmed death-ligand 1
TAM: tumor-associated macrophage
T_CM_: central memory T cell
T_EM_: effector memory T cell
T_EMRA_: terminally-differentiated effector memory T cell
TFR: treatment-free remission
Th1: T helper type 1
TKIs: tyrosine kinase inhibitors
T_N_: naïve T cell
TNF: tumor necrosis factor
Tregs: regulatory T cells
