# Supplementary figures and images for: Flow cytometry and targeted immune transcriptomics identify distinct profiles in patients with chronic myeloid leukemia receiving tyrosine kinase inhibitors with or without interferon-α
Source: J Transl Med. 2020 Jan 3;18:2. doi: 10.1186/s12967-019-02194-x (PMC6941328; doi:10.1186/s12967-019-02194-x)

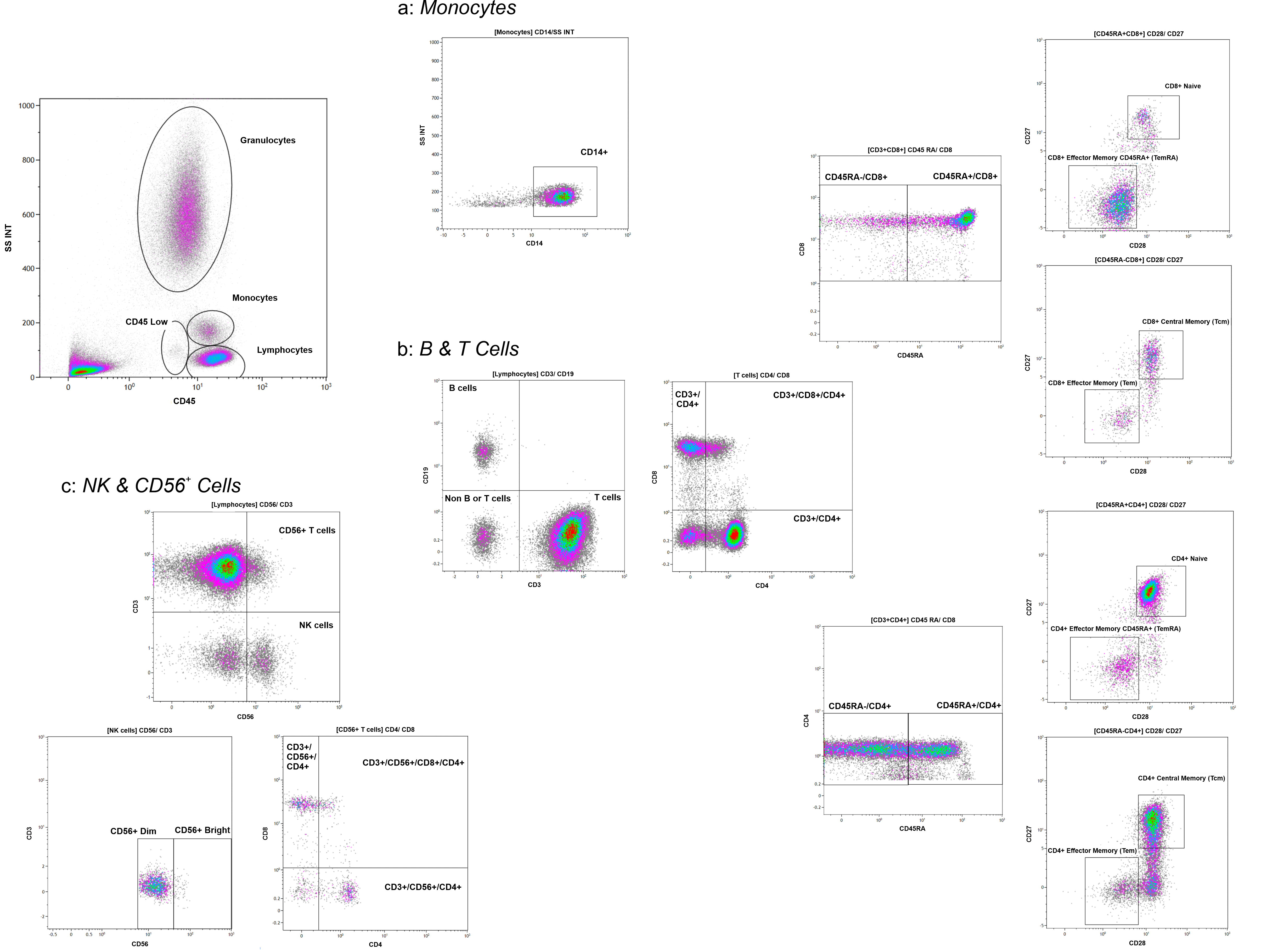

Supplement: Supplementary file 1 — Additional file 1. Gating strategy for the enumeration of peripheral blood populations of the immune system [referred to as the overview of immune system (OVIS) panel]. Leukocyte populations were initially identified based on CD45 expression and side scatter characteristics. CD14 was used as a monocytic marker (A), whereas B and T cells were defined based on the expression of CD19 and CD3, respectively (B). CD4 and CD8 subpopulations were further categorized using CD45RA, CD27 and CD28 staining. (C) NKT cells were identified based on CD56 and CD3 expression, and CD56+ T cells were further subdivided based on CD4 and CD8 expression. [file 12967_2019_2194_MOESM1_ESM.tif]

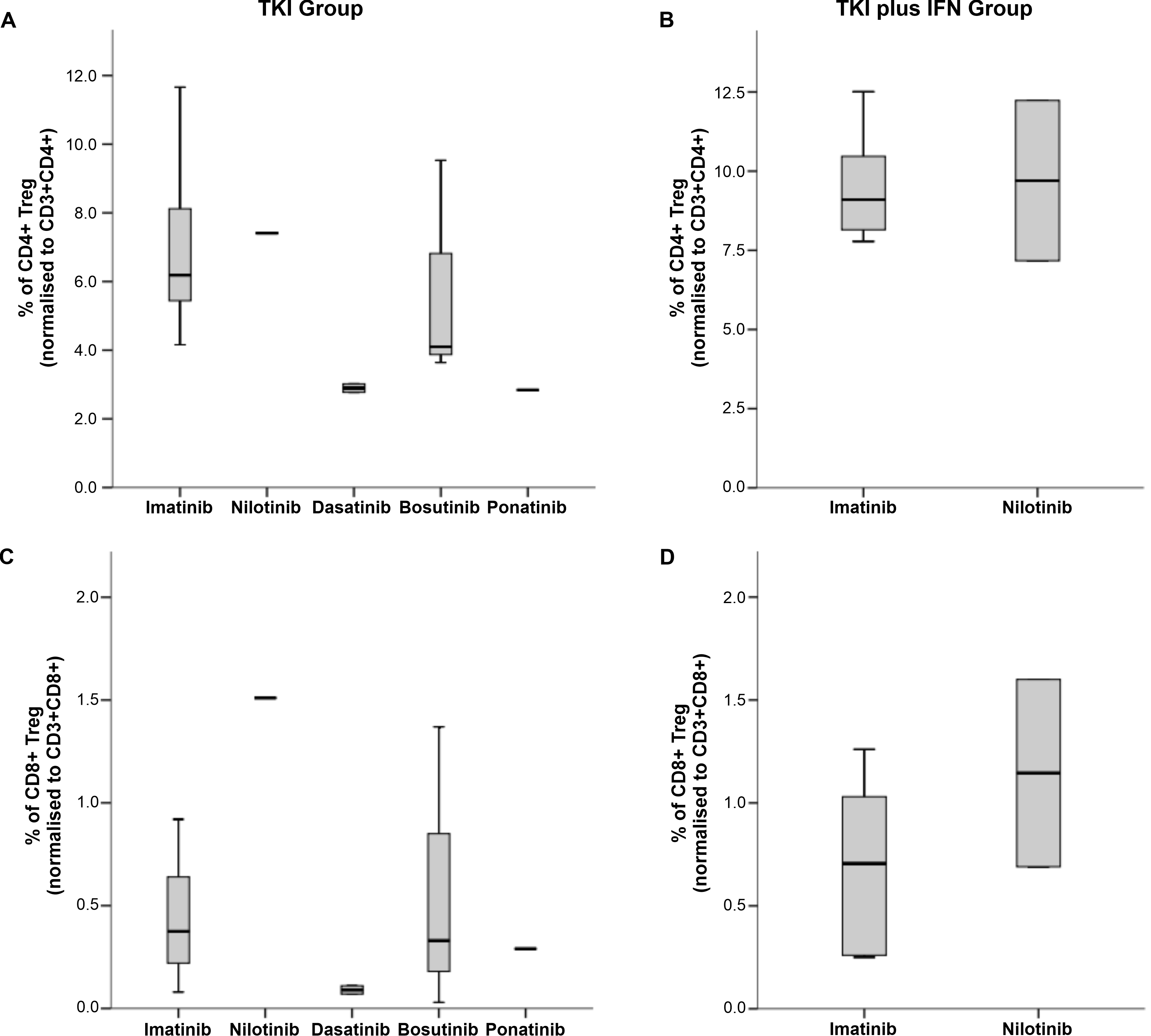

Supplement: Supplementary file 2 — Additional file 2. Frequency of Treg cells in patients with CML receiving imatinib or 2nd generation TKIs. Panels (A) and (B) summarize the frequency of CD4+ Treg cells in patients with CML receiving imatinib (n = 26) or 2nd generation TKIs (n = 1 nilotinib, n = 2 dasatinib, n = 3 bosutinib and n = 1 ponatinib). Panels (C) and (D) depict the frequency of CD8+ Treg cells in the same treatment categories. In the combination treatment group, 6 CML patients were treated with imatinib and 2 CML patients received nilotinib. [file 12967_2019_2194_MOESM2_ESM.tif]

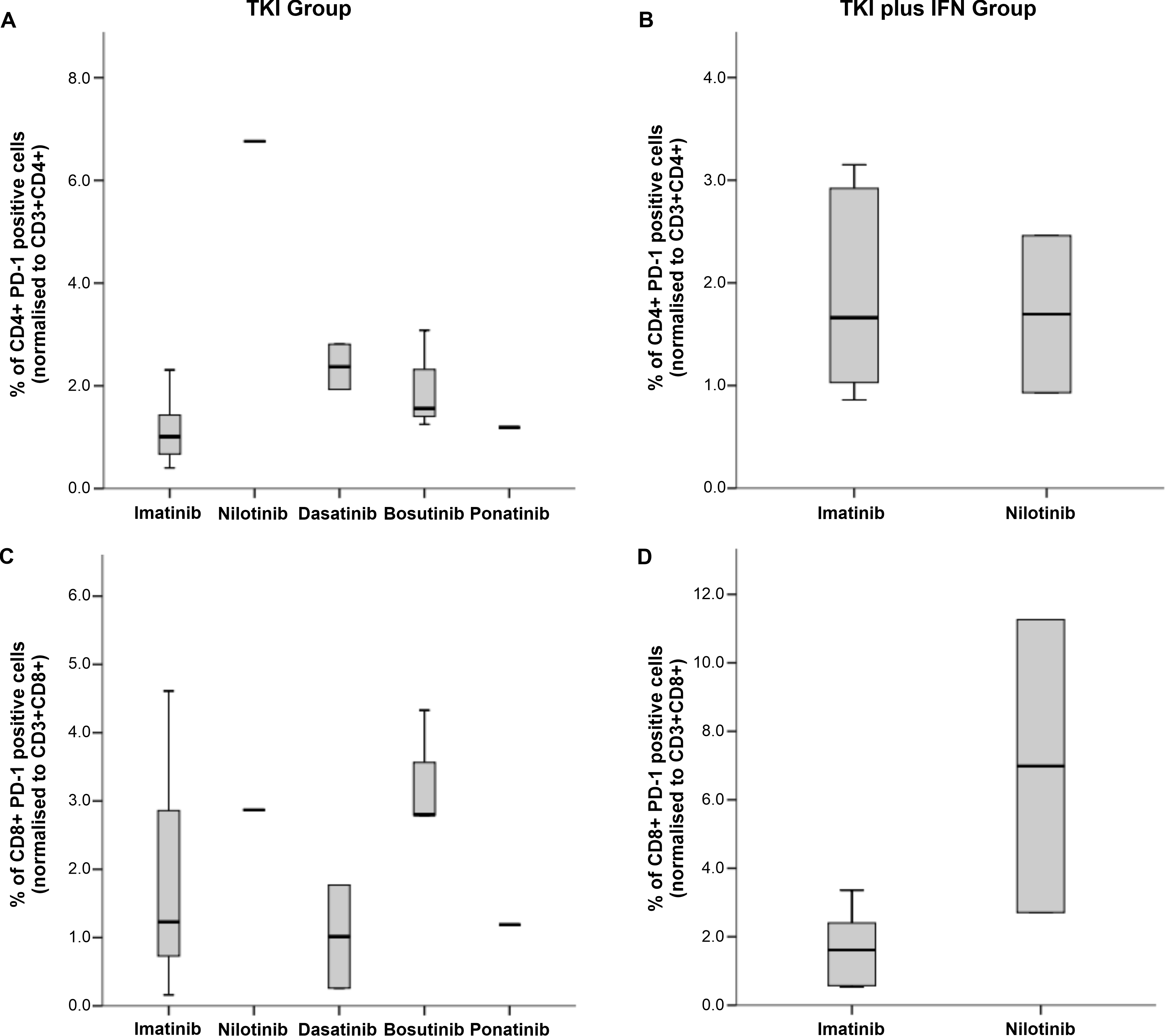

Supplement: Supplementary file 3 — Additional file 3. Programmed death receptor 1 (PD-1) expression in patients with CML receiving imatinib or 2nd generation TKIs. Panels (A) and (B) summarize the frequency of PD-1-expressing CD4+ T cells in patients with CML receiving imatinib (n = 26) or 2nd generation TKIs (n = 1 nilotinib, n = 2 dasatinib, n = 3 bosutinib and n = 1 ponatinib). Panels (C) and (D) depict the frequency of PD-1-expressing CD8+ T cells in the same treatment categories. In the combination treatment group, 6 CML patients were treated with imatinib and 2 CML patients received nilotinib. [file 12967_2019_2194_MOESM3_ESM.tif]

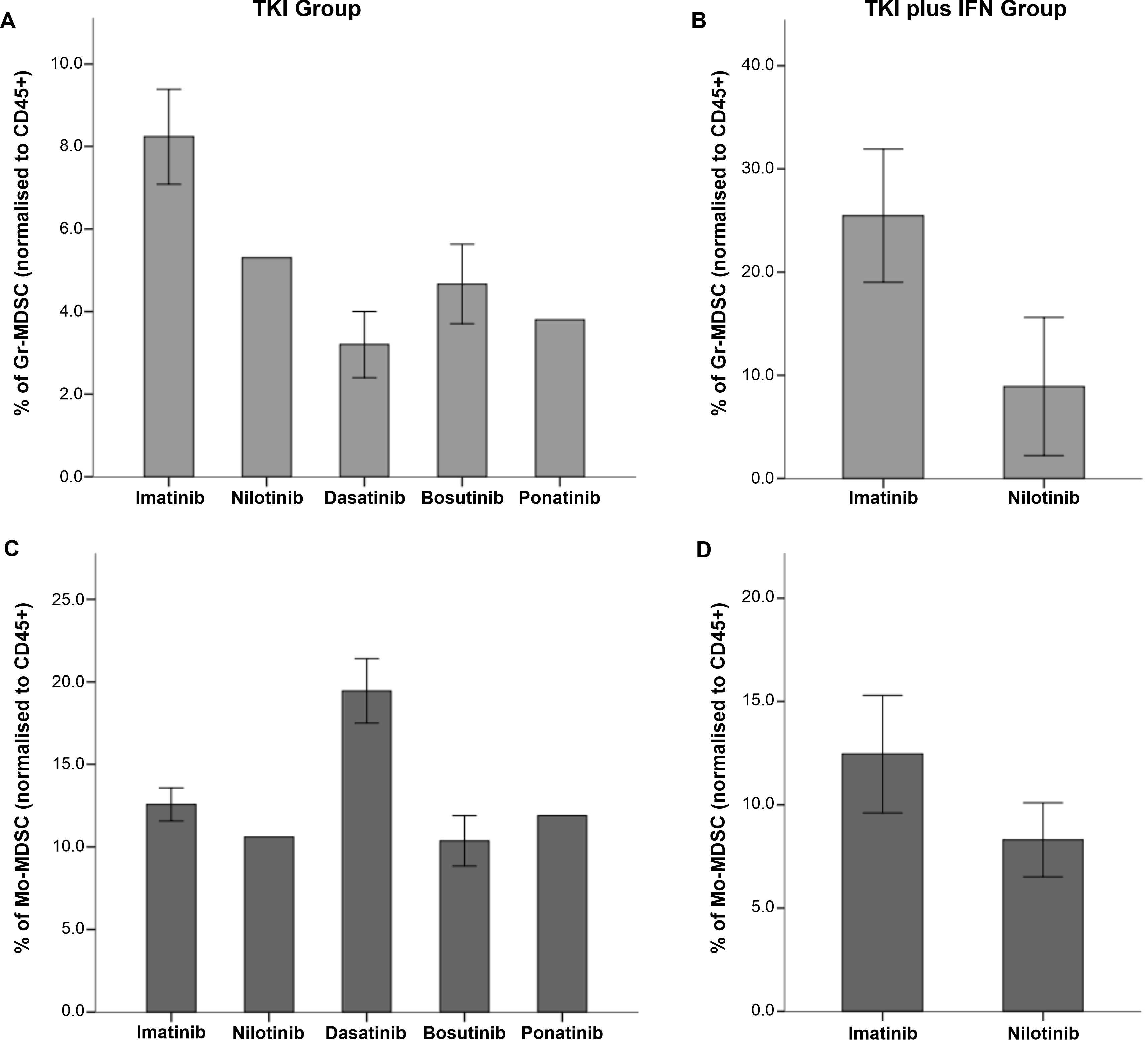

Supplement: Supplementary file 4 — Additional file 4. Frequency of myeloid-derived suppressor cells (MDSCs) in patients with CML receiving imatinib or 2nd generation TKIs. Panels (A-C) and (B-D) summarize the frequency of Gr-MDSCs and Mo-MDSCs, respectively, in patients with CML receiving imatinib (n = 26) or 2nd generation TKIs (n = 1 nilotinib, n = 2 dasatinib, n = 3 bosutinib and n = 1 ponatinib). In the combination treatment group, 6 CML patients were treated with imatinib and 2 CML patients received nilotinib. [file 12967_2019_2194_MOESM4_ESM.tif]
