# Supplementary material for: Flow cytometry and targeted immune transcriptomics identify distinct profiles in patients with chronic myeloid leukemia receiving tyrosine kinase inhibitors with or without interferon-α
Source: J Transl Med. 2020 Jan 3;18:2. doi: 10.1186/s12967-019-02194-x (PMC6941328; doi:10.1186/s12967-019-02194-x)
Supplement: Supplementary file 5 — Additional file 5. List of differentially expressed immune genes when comparing CML patients treated with TKIs plus IFN-α and patients receiving TKIs alone. The differentially expressed genes (fold change > 4 or < 2) are ranked by corrected p value. Data were analyzed using the nSolver™ software package, version 4.0 (NanoString Technologies Inc., Seattle, WA). [file 12967_2019_2194_MOESM5_ESM.docx]

| **Gene symbol** | **Fold change (Log_2_)** | **Std error  (Log_2_)** | **Confidence Interval (Log_2_)** | **Fold change (Linear)** | **Confidence Interval (Linear)** | ***p value*** | **probe.ID** |
| --- | --- | --- | --- | --- | --- | --- | --- |
| *IFIT1* | 6.30 | 0.80 | (4.73 - 7.86) | 78.7 | (26.5 - 233) | 3.05E-07 | NM_001548.3:1440 |
| *ISG15* | 5.76 | 0.63 | (4.53 - 6.99) | 54.1 | (23.1 - 127) | 3.26E-08 | NM_005101.3:305 |
| *SIGLEC1* | 4.97 | 0.64 | (3.72 - 6.23) | 31.4 | (13.2 - 75.0) | 3.72E-07 | NM_023068.3:5165 |
| *OAS3* | 4.61 | 0.48 | (3.67 - 5.54) | 24.4 | (12.8 - 46.5) | 1.47E-08 | NM_006187.2:4980 |
| *IFIT2* | 4.33 | 0.76 | (2.85 - 5.81) | 20.1 | (7.21 - 56.2) | 1.96E-05 | NM_001547.4:1995 |
| *SERPING1* | 4.00 | 0.49 | (3.04 - 4.96) | 16.0 | (8.21 - 31.2) | 1.89E-07 | NM_000062.2:305 |
| *MX1* | 3.95 | 0.47 | (3.03 - 4.86) | 15.4 | (8.18 - 29.1) | 1.11E-07 | NM_002462.2:1485 |
| *IFI35* | 3.25 | 0.66 | (1.95 - 4.54) | 9.48 | (3.87 - 23.3) | 1.12E-04 | NM_005533.3:415 |
| *DDX58* | 3.22 | 0.64 | (1.97 - 4.46) | 9.30 | (3.92 - 22.1) | 8.16E-05 | NM_014314.3:2130 |
| *TNFSF10* | 3.20 | 0.57 | (2.09 - 4.31) | 9.18 | (4.25 - 19.8) | 2.35E-05 | NM_003810.2:115 |
| *CXCL10* | 2.89 | 0.62 | (1.67 - 4.11) | 7.41 | (3.18 - 17.3) | 2.04E-04 | NM_001565.1:40 |
| *IRF7* | 2.77 | 0.47 | (1.85 - 3.70) | 6.83 | (3.59 - 13.0) | 1.48E-05 | NM_001572.3:1763 |
| *IFITM1* | 2.18 | 0.52 | (1.16 - 3.20) | 4.53 | (2.23 - 9.22) | 5.71E-04 | NM_003641.3:482 |
| *CD38* | 2.13 | 0.34 | (1.51 - 2.76) | 4.39 | (2.85 - 6.76) | 2.65E-06 | NM_001775.2:460 |
| *BST2* | 2.11 | 0.27 | (1.58 - 2.65) | 4.33 | (2.99 - 6.27) | 3.73E-07 | NM_004335.2:560 |
| *ISG20* | 2.09 | 0.37 | (1.36 - 2.82) | 4.26 | (2.57 - 7.06) | 2.44E-05 | NM_002201.4:358 |
| *IL1RN* | 2.05 | 0.64 | (0.80 - 3.30) | 4.14 | (1.74 - 9.84) | 4.80E-03 | NM_000577.3:480 |
| *KIR3DL2* | -1.24 | 0.38 | (-2.00 - -0.49) | 0.42 | (0.25 - 0.71) | 4.78E-03 | NM_006737.2:884 |
| *KLRB1* | -1.27 | 0.32 | (-1.91 - -0.64) | 0.41 | (0.27 - 0.64) | 9.53E-04 | NM_002258.2:85 |
| *FEZ1* | -2.09 | 0.46 | (-2.99 - -1.19) | 0.23 | (0.13 - 0.44) | 2.79E-04 | NM_005103.4:426 |
| *KIR_Activating_Subgroup_2* | -2.60 | 0.64 | (-3.86 ­- -1.35) | 0.17 | (0.07 - 0.39) | 8.02E-04 | NM_014512.1:718 |
| *KIR_Activating_Subgroup_1* | -3.38 | 0.84 | (-5.03 - -1.73) | 0.10 | (0.03 - 0.30) | 9.11E-04 | NM_001083539.1:1146 |
|  | | | | | | | |

**Additional File 5 – Differentially expressed genes**
